# Sexual conflict and social networks in bed bugs: effects of social experience

**DOI:** 10.1093/beheco/arae030

**Published:** 2024-04-17

**Authors:** Janice L Yan, Jack R Rosenbaum, Selena Esteves, Maggie L Dobbin, Reuven Dukas

**Affiliations:** Animal Behaviour Group, Department of Psychology, Neuroscience & Behaviour, McMaster University, 1280 Main Street West, Hamilton, ON, L8S 4K1, Canada; Animal Behaviour Group, Department of Psychology, Neuroscience & Behaviour, McMaster University, 1280 Main Street West, Hamilton, ON, L8S 4K1, Canada; Animal Behaviour Group, Department of Psychology, Neuroscience & Behaviour, McMaster University, 1280 Main Street West, Hamilton, ON, L8S 4K1, Canada; Animal Behaviour Group, Department of Psychology, Neuroscience & Behaviour, McMaster University, 1280 Main Street West, Hamilton, ON, L8S 4K1, Canada; Animal Behaviour Group, Department of Psychology, Neuroscience & Behaviour, McMaster University, 1280 Main Street West, Hamilton, ON, L8S 4K1, Canada

**Keywords:** sexual conflict, social experience, sexual competence, social network analysis, bed bugs, *Cimex lectularius*

## Abstract

Living in groups can provide essential experience that improves sexual performance and reproductive success. While the effects of social experience have drawn considerable scientific interest, commonly used behavioral assays often do not capture the dynamic nature of interactions within a social group. Here, we conducted 3 experiments using a social network framework to test whether social experience during early adulthood improves the sexual competence of bed bugs (*Cimex lectularius*) when placed in a complex and competitive group environment. In each experiment, we observed replicate groups of bed bugs comprising previously socialized and previously isolated individuals of the same sex, along with an equal number of standardized individuals of the opposite sex. Regardless of whether we controlled for their insemination history, previously isolated males mounted and inseminated females at significantly higher rates than previously socialized males. However, we found no evidence of social experience influencing our other measures of sexual competence: proportion of mounts directed at females, ability to overcome female resistance, and strength of opposite-sex social associations. We similarly did not detect effects of social experience on our female sexual competence metrics: propensity to avoid mounts, rate of successfully avoiding mounts, opposite-sex social association strength, and rate of receiving inseminations. Our findings indicate that early social experience does not improve sexual competence in male and female bed bugs.

## Introduction

Social experience can drastically shape the physiology (Sachser and Lick 1991; Crews et al. 1997), brain-structure (Crews et al. 1997; Champagne and Curley 2005), cognitive abilities (Chapman et al. 2008; Taborsky et al. 2012), and behavior (Polt and Hess 1966; Lihoreau et al. 2009) of animals. Experience with same-sex conspecifics can provide crucial information about local levels of competition, allowing for plastic responses that increase an individual’s fitness (Gage and Baker 1991; Bretman et al. 2010). Experience with same-sex conspecifics can also be essential for learning skills related to courtship and mating. For example, male zebra finches (*Taeniopygia guttata*) require the presence of other adult males during development to adequately learn courtship singing and develop a preference for opposite-sex stimuli, two essential components of mating success in this species (Immelmann 1969; Adkins-Regan and Krakauer 2000).

Opposite-sex interactions can similarly be crucial as they provide opportunities for sexual experience leading to better performance in future sexual encounters (Pérez-Staples et al. 2010; Milonas et al. 2011; Orihuela and Aguirre 2011). Even failed mating attempts can provide individuals with valuable feedback from unreceptive individuals, which can improve sexual pursuit strategies and aid in narrowing the range of sexual partners one pursues (Dukas 2004, 2005). The overall importance of social experience is further exemplified by the fact that social isolation has been shown to alter an individual’s subsequent behavior and fitness in mammals (Harlow et al. 1965), birds (Baron and Kish 1960), fishes (Taborsky et al. 2012; Hesse and Thünken 2014), and insects (Baxter and Dukas 2017).

Despite the well-documented effects of social isolation across taxa (Gerall et al. 1967; Kim and Ehrman 1998; Cacioppo and Hawkley 2009), prior studies that experimentally manipulate social experience largely assess its influence using simple mating and behavioral assays on either individual behavior or dyadic interactions (Sakata et al. 2002; Arnold and Taborsky 2010; Guevara-Fiore 2012; Řežucha and Reichard 2014; Lehtonen et al. 2016; Favati et al. 2021). While highly informative, these approaches do not reflect the full range of social and environmental pressures that most animals face under natural settings. For example, commonly used behavioral assays typically do not capture challenges associated with mate search, competition from rivals, and the possibility for females to successfully evade undesired sexual pursuit. Furthermore, while there has been ample research on how experience shapes male courtship and mating strategies (Dukas 2005; Bailey et al. 2010; Jordan and Brooks 2012; Rodríguez et al. 2013; Řežucha and Reichard 2014; Lehtonen et al. 2016), few studies have investigated the effects of social experience using a sexual conflict framework to examine whether prior experience influences females’ tendency and ability to evade costly pursuit (but see Killen et al. 2016 for a notable exception). Yet, seeing as sexual conflict and male harassment of females is pervasive among sexually reproducing animals (Parker 1979; Clutton-Brock and Parker 1995; Chapman 2006), we may expect females to exhibit behavioral plasticity in response to prior exposure to conspecifics. Overall, our current understanding of how social experience influences both males’ and females’ subsequent behaviors remains limited. Therefore, we sought to address fundamental knowledge gaps by examining how the social environment in early adulthood shapes the subsequent sexual competence of both males and females in a naturalistic, complex, and competitive environment using bed bugs (*Cimex lectularius*) as a model system.

Bed bugs are a frequently cited example of extreme sexual conflict as they obligately reproduce through traumatic insemination, whereby males pierce and deposit sperm directly into females’ abdomens. Likely owing to the energetic costs of wound healing and increased frequency of infection, realistic rates of repeated traumatic insemination have been shown to dramatically reduce both longevity and lifetime reproductive output of female bed bugs (Stutt and Siva-Jothy 2001; Yan et al. under review). Thus, while males should try to maximize number of inseminations, females are under selective pressure to evade excess inseminations. Bed bugs also display moderate social behavior. They are typically found in mixed-sex aggregations within tight crevasses and possess volatile, non-volatile, and tactile cues that facilitate social attraction (Carayon 1966; Reinhardt and Siva-Jothy 2007; Siljander et al. 2007, 2008; Yan and Dukas 2022). Combined, these sexual and social features of bed bugs make them an ideal model for examining how one’s prior social environment can shape sexual competence as group-living may lead to the acquisition of information and experience that can improve the outcome of subsequent sexual interactions.

In addition to documenting the individuals involved and the outcomes of sexual interactions, we also constructed social networks based on hourly scans of bed bugs’ social partners. Occupying more central network positions in mixed-sex (Ryder et al. 2008; Oh and Badyaev 2010; Formica et al. 2012) and opposite-sex association networks (Godfrey et al. 2012; Sabol et al. 2020; Beck et al. 2021; Dunning et al. 2023) has been positively linked to mating success and fitness in several taxa. However, the traits and environmental factors that determine an individual’s position in a social network remain poorly understood. There are currently only a small handful of studies assessing the relationship between social experience and future network position (McDonald 2007; Crailsheim et al. 2020; Kurvers et al. 2020), and even fewer that experimentally manipulate experience to explicitly test its effects on various network metrics (Riley et al. 2018; Brandl et al. 2019; Bentzur et al. 2021). To address these gaps, we examined how prior experience with conspecifics influences the strength of individuals’ opposite-sex associations. We focused on opposite-sex social associations as we believed they would represent a crucial aspect of bed bugs’ social environments given that they exhibit extreme polyandry and sexual conflict. Since prior animal network studies have documented strong links between social network position and several measures of reproductive success (McDonald et al. 2019; Beck et al. 2021; Dunning et al. 2023), we expected strong connections to opposite-sex conspecifics to reflect males’ ability to locate females and retain continued access to insemination opportunities, and females’ inability to successfully find refuge from continued sexual pursuit from males.

We first examined how social experience shapes the sexual competence of males by generating groups of previously isolated males, previously socialized males, and new, unfamiliar females and observing all sexual and social interactions within complex arenas. This approach created a dynamic setting where experienced and inexperienced males directly competed with one another for access to females in an environment where females could readily escape insemination attempts. We predicted that, compared to previously isolated males, socially experienced males would direct a higher proportion of mounts at females as opposed to males, be better at overcoming female avoidance, form stronger opposite-sex social network associations, and achieve higher overall insemination rates. Second, we tested the effects of social experience on female bed bugs’ sexual competence. Specifically, we examined whether previously socialized females were more adept at avoiding costly inseminations compared to previously isolated females. Since we expected females in the social treatment to gain relevant experience in avoiding persistent sexual pursuit in a complex environment with several males, we predicted that previously socialized females would attempt to evade more mounts, successfully evade more mounts, form weaker opposite-sex social network associations, and be inseminated at lower overall rates compared to previously isolated females.

## Methods

### Ethical note

Our research complied with all laws and did not require ethics committee approval. While we do not require ethics approval, we treat our subjects in accordance with strict animal ethics standards under the assumption that they experience emotion in general and pain in particular.

### Study population and maintenance

Our colony of bed bugs (*Cimex lectularius*) was derived from 4 natural infestations collected in Southern Ontario between October 2019 and January 2020. We housed our colony in 2 large 54 × 40 × 40 cm plastic storage bins kept at 27 ± 0.5 °C at 60% relative humidity with lights off at 0800 h and on at 1600 h. In each plastic bin, we kept bed bugs in 85 mL spice jars, each containing several strips of folded filter paper to provide a rough surface for walking and oviposition. Each jar housed approximately 50 to 150 bed bugs of similar life stages, with adults always housed with other adults. Every week, we fed the colony during their dark photoperiod under red light with defibrinated rabbit blood (Hemostat Laboratories, Dixon, CA) and using a Hemotek membrane-feeding system (Discovery Workshops, Accrington, UK). In all experiments, we generated virgin bed bugs by individually isolating recently fed fifth-instar bed bugs and grouping them into same-sex groups once they emerged as adults. We marked all focal bed bugs one at a time by briefly anesthetizing them with CO_2_, then fastening them onto a wedge-shaped sponge with a single strand of hair. Once secured, we used a toothpick to apply a unique number ID to each bed bug using white paint from a Sharpie oil-based paint marker.

### Experiment 1: Effect of social and sexual experience on male sexual competency

#### Experience phase

**Fig. 1.**
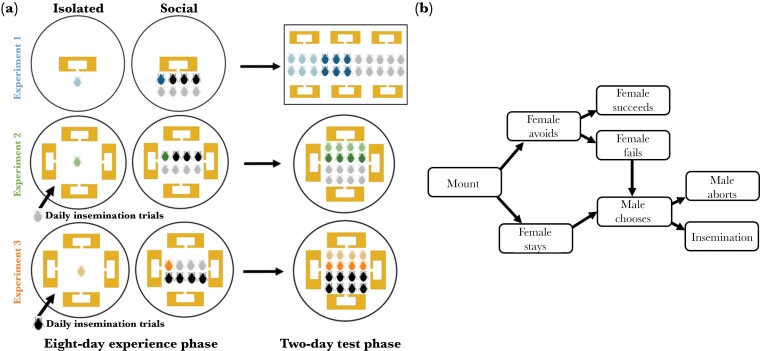
(a) Schematic overview of experimental designs. In Experiment 1, we tested isolated (light blue) vs. socialized (dark blue) males in a competitive environment following an 8-d experience phase. In Experiment 2, we tested isolated (light green) vs. socialized (dark green) males but with additional daily insemination trials for isolated males to control for insemination experience. In Experiment 3, we tested isolated (light orange) vs. socialized (dark orange) females and again controlled for insemination experience. For all experiments, gray bed bugs represent standard age-matched virgin females and black bed bugs represent standard age-matched virgin males. (b) Flowchart illustrating how we scored sexual interactions and their outcomes.

All 3 of our experiments consisted of an 8-d long experience phase followed by a 2-d test phase. In Experiment 1, we assessed the general effect of differential social environments on focal male bed bugs. This meant that males from social vs. isolated treatments differed in their exposure to male and female conspecifics and thus also differed in insemination status prior to the observation phase. To generate focal male bed bugs, we marked and fed a group of 1-d-old virgin males, then randomly assigned 6 males to the social treatment and 6 males to the isolated treatment. We placed each social focal male in a 15 cm petri dish lined with filter paper along with 3 age-matched virgin males and 4 age-matched virgin females (Fig. 1a). We placed each isolated focal male in an identical arena as the social males but with no other bed bugs (Fig. 1a). In each arena, we also placed a wooden shelter constructed from a 25 × 75 × 3 mm balsa wood slat segment covered with a glass microscope slide. Each wood segment contained a 15 × 30 mm cavity with a narrow 5 mm entrance (Fig. 1a). Our previous studies have shown that bed bugs readily seek refuge, form aggregations, and engage in sexual interactions within such shelters (Yan et al. under review; Yan and Dukas 2022). We also generated age-matched virgin females for focal males to interact with during the test phase by group-housing 24 females in 15 cm petri dish arenas with 4 balsa wood shelters. Since bed bug reproductive behavior is closely tied to feeding, we fed all focal males on the last day of the experience phase by briefly (< 15 min) grouping individuals by treatment, then returning each bed bug to its respective arena. We also fed all females that were used in the experiment on the last day of the experience phase.

#### Test phase

Following the 8-d experience phase, we placed the 6 social males and 6 isolated males along with twelve new individually marked, age-matched virgin females into a 34.5 × 23.5 × 15 cm Plexiglass rectangular arena (Fig. 1). Each social male was obtained from a different social experience arena so that all males were unfamiliar with each other. The arena was lined with filter paper and fitted with 6 brand new balsa wood shelters, identical to those used in the experience phase. We released the bed bugs at the center of the arena at the start of their dark photoperiod (0800 h), and an observer continuously scored all sexual interactions in real time for the entire duration of their dark photoperiod (0800–1600 h) for 2 consecutive days. We opted not to document interactions during the light photoperiod as bed bugs are nocturnal and, therefore, largely inactive during the light phase (Mellanby 1939; Romero et al. 2010). Furthermore, our previous data indicate that greater than 85% of sexual interactions occur during bed bugs’ dark photoperiod (Yan and Dukas 2022). We recorded all mounts and inseminations along with the identities of the individuals involved in each interaction and the outcomes of each interaction according to the flowchart in Fig. 1b. We ensured that observers were always blind to male treatment.

Both mounting and traumatic insemination are highly stereotyped and distinctive behaviors in bed bugs. A mount consists of a male “jumping” onto another bed bug and then dismounting typically within 5 s (Stutt and Siva-Jothy 2001). Male bed bugs are known to frequently mount but rarely inseminate other males (Rivnay 1933; Ryne 2009). An insemination is characterized by a male mounting a female, then remaining securely attached with his abdomen curled underneath the female’s right abdomen for up to 5 min (1 to 5 min, Carayon 1966, p. 103; 30–300 s, Figure 2 in Siva-Jothy and Stutt 2003). In a data set including 193 insemination durations recorded in our laboratory for another experiment, the average ± 1 SD insemination duration was 102.4 ± 53.9 s, and the range was 18 to 406 s, with only one insemination lasting less than 20 s (Yan and Dukas 2022). Therefore, we chose 20 s as the minimum duration for a mount to be considered an insemination. If males appeared to voluntarily dismount a female before getting into the insemination position or within the first 20 s of being in the insemination position, we considered the mount to be aborted by the male. We scored female avoidance attempts based on whether females attempted to run away or displayed the refusal posture in response to a mount (Siva-Jothy 2006) and avoidance success based on whether the female successfully prevented the mounting male from assuming insemination position.

In addition to live, continuous scoring of sexual interactions, we also examined bed bugs’ social associations by performing scans where we documented the location of each bed bug. Because the roofs of our shelters were constructed with clear microscope slides, we could readily determine the location of each bed bug without causing disruptions. We later used this information to construct networks based on shared shelter use. We conducted a scan at the start of each hour of the dark phase for a total of 8 scans on day 1 and 9 scans on day 2. In total, we conducted 6 replicates of the experiment. We documented clear behavioral abnormalities in 2 males from the social treatment, one from replicate one and another from replicate two. Both males were unable to inseminate females. We decided to remove them from the analyses before knowing what treatment they belonged to, resulting in a final sample size of 34 for the social treatment and 36 for the isolated treatment.

### Experiment 2: Effect of social experience on male sexual competency, controlling for insemination status

#### Experience phase

Next, we tested the effect of social experience on males while controlling for insemination status. We generated focal male bed bugs by marking and feeding a group of 1-d-old virgin males, then randomly assigned 4 males to the social treatment and 4 males to the isolated treatment. We placed each social focal male in a 150-mm petri dish lined with filter paper along with 3 age-matched virgin males and 4 age-matched virgin females. We placed each isolated focal male in an identical arena as the social males but with no other bed bugs. Here, the experience phase arenas differed from Experiment 1 in that each arena contained 4 instead of one wooden shelter. We decided to include more shelters in each arena to better mimic naturalistic environments where individuals may have to move amongst multiple refuges to locate sexual or social partners and to ensure individual experiences were relevant for the observation phase, where we also provided bed bugs with multiple shelters. To accommodate the greater number of shelters, we reduced the dimensions of the wooden slat from 75 mm long to 40 mm long by trimming off excess wood. The size of the actual shelter cavity and entrance remained the same.

To roughly equalize the insemination status of social and isolated focal males prior to the observation phase, we conducted controlled and brief insemination trials for isolated males on 7 out of the 8 experience phase days. We selected 7 out of 8 d to mirror previously observed rates of traumatic insemination in bed bugs (Johnson 1941; Stutt and Siva-Jothy 2001; Yan and Dukas 2022). For example, when placed in a semi-naturalistic environment with ample room and protective shelters, the average rate of traumatic insemination in bed bugs was 0.89 inseminations per day (Yan and Dukas 2022). We conducted insemination trials between 1000 and 1200 h each day by placing each male from the isolated treatment in small, 30 mm petri dishes lined with filter paper along with a single, age-matched female. We continuously observed each pair of bed bugs until the male dismounted the female following insemination and then placed each male back into his respective arena. Most insemination trials lasted less than 5 min. If males did not inseminate females within 10 min, we added a second age-matched female to their arena. If males still did not inseminate either of the two females after another 10 min, we returned them to their experience phase arena without completing an insemination. This happened in 12/168 trials and never more than once for the same male; therefore, we continued to use males who missed an insemination in the experiment. Lastly, to ensure that males from the social treatment received equivalent levels of handling, we also briefly removed social males from their experience phase arenas and placed them into 30 mm petri dishes for approximately the same duration of a typical insemination trial before returning them to their respective dishes. On the final day of the experience phase, we fed all focal males by briefly grouping individuals by treatment, then returned each bed bug to their respective arena. We also generated age-matched virgin females for focal males to interact with during the test phase by group-housing 24 females in 15 cm petri dish arenas with 4 balsa wood shelters and fed these females on the last day of the experience phase.

#### Test phase

Following the 8-d experience phase, we placed 4 social males and 4 isolated males along with 8 new individually marked age-matched virgin females into a new 150 mm petri dish arena with 4 wooden shelters, identical to the arenas bed bugs were kept in during their experience phase. We opted to use this arena instead of the large, rectangular Plexiglass arena used in Experiment 1 because we wanted the physical environment to remain consistent between the experience and test phases. Otherwise, all other aspects of the test phase in Experiment 2 were identical to Experiment 1. In total, we conducted 6 replicates of the experiment resulting in a final sample size of 24 males per treatment.

### Experiment 3: Effect of social experience on female sexual competency, controlling for insemination history

#### Experience phase

Here, we tested the effect of social experience on females’ propensity and ability to evade costly inseminations while controlling for female insemination status. We used an identical protocol to Experiment 2 except the focal individuals were now female instead of male. Briefly, we marked and fed focal female bed bugs and then randomly assigned 4 females to the social treatment and 4 females to the isolated treatment. We housed social females with 3 age-matched virgin females and 4 age-matched virgin males. To roughly equalize the insemination status of social and isolated focal females prior to the observation phase, we conducted controlled and brief insemination trials for isolated females on 7 out of the 8 experience phase days. If females were not inseminated within 10 min, we added a second age-matched male to their arena. If either of the two males still did not inseminate the focal female after another 10 min, we returned the female to their experience phase arena without completing an insemination. This occurred in 7/168 trials and never more than once for the same female, thus females that received one fewer insemination were still used in the experiment. On the final day of the experience phase, we fed all the focal females by briefly grouping individuals by treatment, then returned each bed bug to their respective arena. To verify that females of the social treatment encountered males frequently, we videorecorded the interactions of 4 social females with males over a single 8-h dark photoperiod in replicate two using a Canon VIXIA HF R800 camera. We only examined the dark photoperiod because bed bugs are mostly inactive during their light phase (Mellanby 1939; Romero et al. 2010). On average, these social females encountered males 12.5 times, were mounted by males 6.25 times, and were inseminated by males 1.25 times over the course of a single dark photoperiod. Lastly, we generated age-matched virgin males for focal females to interact with during the test phase by group-housing 24 males in 15 cm petri dish arenas with 4 balsa wood shelters and fed these males on the last day of the experience phase.

#### Test phase

Following the 8-d experience phase, we placed 4 isolated females and 4 social females with 8 new individually marked, age-matched, males into a new 150 mm petri dish arena lined with 4 wooden shelters, identical to the arenas bed bugs were kept in during their experience phase. We ensured that all the males had mated one day prior to the observation phase so that they had some prior sexual experience. The test phase for this experiment was conducted identically to that of Experiment 2 except that we did not record male-male mounts as these interactions did not directly involve the focal females. In total, we conducted 6 replicates of the experiment resulting in a final sample size of 24 females per treatment.

### Statistics

We completed all our analyses in R version 4.1.1 (R Core Team 2021) and used the package “glmmTMB” v.1.1.2.2 (Brooks et al. 2017) to construct all our GLMMs (Generalized Linear Mixed Models). We verified all model fits by visually inspecting plots of model residuals using the “DHARMa” package (Hartig 2018) and assessed the significance of fixed effects using the *Anova* function from the “car” package (Fox and Weisberg 2019). All our GLMMs included treatment, day, and their interaction as fixed factors and replicate and individual ID as random factors. Our analyses were identical for Experiments 1 and 2 as both assessed the effect of social experience on males. For each of these two experiments, we first constructed a GLMM with a binomial distribution to examine whether social experience affected how often males mounted other males as opposed to females. To construct the response variable, we used the *cbind()* function to combine the number of mounts each male directed at males and the number of mounts each male directed at females. Next, we fit a binomial GLMM for each male experiment to examine whether females escaped from previously isolated males at higher rates. Specifically, we constructed the response variable by using *cbind()* to combine mounts that females attempted to but failed to avoid and mounts that females successfully avoided for each male. Then, we constructed a GLMM with a number of inseminations secured per male as the response variable for each male experiment. These models were fitted with a Poisson distribution. Since isolated males secured more inseminations than socialized males in both Experiments 1 and 2 despite showing no differences in our various sexual competency metrics, we additionally tested for differences in total mounts performed to see if males from the two treatments differed in sexual motivation. To do this, we constructed GLMMs with total mounts performed by each male (regardless of which sex they were directed at) as the response variable. For Experiment 1, we used a negative binomial distribution since a Poisson model resulted in significant deviations from normality, and for Experiment 2, we used a Poisson distribution.

For Experiment 3, which assessed the effects of social experience on females, we first tested whether social experience affected the rate at which females attempted to avoid mounts. To do this, we ran a GLMM using a binomial distribution and the *cbind()* function to combine a number of attempted avoidances and a number of mounts where females did not attempt to avoid as the response variable. We next assessed the avoidance success rate by fitting a GLMM using a binomial distribution and the *cbind()* function to combine a number of successful mount avoidances and a number of failed mount avoidances as the response variable. Then, we tested number of inseminations received using a GLMM with a Poisson distribution. Because isolated females were inseminated more frequently than social females on Day 1, despite showing no differences in their propensity or ability to escape mounts, we additionally tested if females were initially mounted by males at different rates. We tested this using a GLMM with a number of mounts received as the response variable and a negative binomial distribution since a Poisson model violated assumptions of homogenous variance. Lastly, we also additionally examined rates of males terminating mounts directed at isolated vs. social females. To do this, we used a GLMM with a binomial distribution using *cbind* to combine mounts directed at each female that males terminated and mounts directed at each female where males had the opportunity to terminate but did not. For all of our statistical models, we used the package “emmeans” to further test whether the effect of social experience differed between Days 1 and 2 of the test phases if treatment-by-day interactions were statistically significant.

### Social network analyses

We constructed social networks in R using the “igraph” package (Csardi and Nepusz 2006), where edges represented association indices between dyads based on how often two individuals occupied the same shelter during hourly scans. Specifically, we used the simple ratio index to calculate association indices, which is recommended when all individuals can be reliably identified during sampling periods (Hoppitt and Farine 2018). We then eliminated same-sex connections from association matrices to generate networks that selectively captured opposite-sex associations. Next, we extracted strength values from these opposite-sex networks for each individual. Strength is equivalent to the sum of all edge weights connected to a node and in our networks, represents how often and with how many opposite-sex individuals a bed bug was associated with through shared shelter use.

To test whether social versus isolated individuals differed in opposite-sex network strength, we used an LMM combined with a permutation test for each of the 3 experiments. Each LMM included strength as the response variable, treatment as a fixed factor, and replicate as a random factor. Because network measures are inherently non-independent (Croft et al. 2011; Farine and Whitehead 2015), we performed a node-label permutation test for each experiment by shuffling and redistributing individuals of the focal sex amongst their node positions. For example, in Experiment 1, which assessed the effect of experience on males, we shuffled the network positions of the twelve males in each replicate, while in Experiment 3, we shuffled females amongst their network positions. This type of randomization, where individuals of the focal sex are shuffled and randomly redistributed between the two treatment groups, results in social networks representing the null hypothesis that treatment has no bearing on social network position. By performing 1000 iterations of this network randomization process per experiment, we were able to compare model coefficients from observed networks to a distribution of model coefficients representing the null hypothesis that social experience has no bearing on the strength of one’s opposite-sex associations. Such permutation tests are the most widely used approach to control for statistical non-independence between individuals from the same social network ([Bibr CIT0024][Bibr CIT0025]; Farine and Whitehead 2015)

## Results

### Experiment 1: Effect of social and sexual experience on male sexual competency

**Fig. 2.**
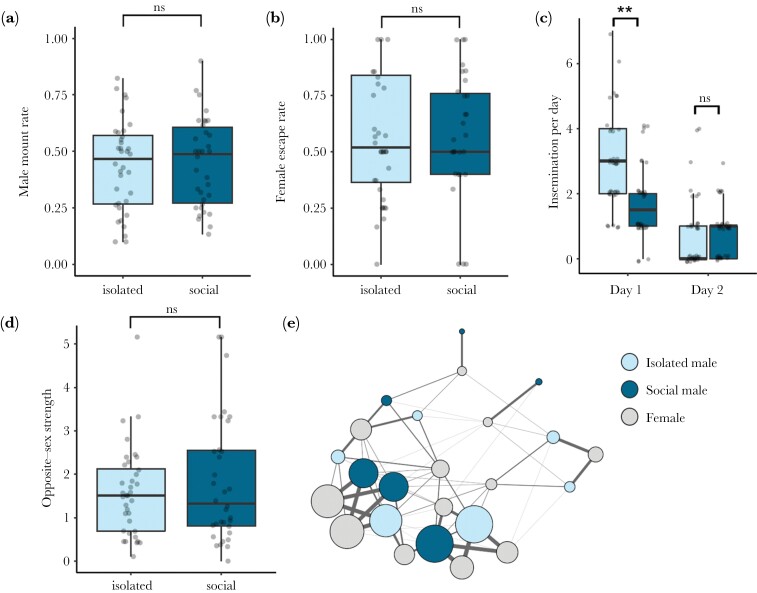
(a) Proportion of mounts males directed towards other males, (b) rate of females successfully evading insemination attempts, (c) insemination rates, and (d) opposite-sex association strength for isolated (*N* = 36) vs. social (*N* = 34) males. Bold horizontal lines indicate the medians, the boxes represent the interquartile range (IQR) between the first and third quartiles, and the whiskers above and below each box represent values within ±1.5 of the IQR. (e) Weighted opposite-sex social association network from one replicate based on patterns of shared shelter use. Node color corresponds to individual treatment. Edge width represents the strength of association between opposite-sex dyads, and node size corresponds to opposite-sex strength (total sum of edge weights). Social networks for all 6 replicates are depicted in Supplementary Fig. S1.

We did not find an effect of prior social and sexual experience on males’ propensity to direct mounts at other males as opposed to females (GLMM: Wald χ^2^_1_ = 0.47, *P* = 0.49; Fig. 2a). Similarly, experience with conspecifics did not generate a significant difference in males’ abilities to prevent females from successfully evading mounts (GLMM: Wald χ^2^_1_ = 0.26, *P* = 0.61; Fig. 2b). However, we did detect a marginally significant treatment by day interaction for the number of inseminations secured (GLMM: Wald χ^2^_1_ = 3.72, *P* = 0.05; Fig. 1c), as isolated males secured significantly more inseminations than social males on Day 1 (*t *= 3.26, *P* < 0.01; Mean ± SE: isolated = 2.97 ± 0.25, social = 1.74 ± 0.19) but not Day 2 (*t = *−0.249, *P* = 0.80; Mean ± SE: isolated = 0.81 ± 0.19, social = 0.85 ± 0.13). Likewise, we detected a significant treatment by day interaction for mounts performed (GLMM: Wald χ^2^_1_ = 11.56, *P* < 0.001), again because isolated males performed more mounts than socialized males on Day 1 (*t* = 4.18, *P* < 0.001; Mean ± SE: isolated = 19.31 ± 2.14, social = 10.32 ± 1.24) but not Day 2 (*z* = −0.83, *P* = 0.41; Mean ± SE: isolated = 5.75 ± 1.15, social = 6.62 ± 0.75). We also found significant day effects, with males from both treatments performing more inseminations (GLMM: Wald χ^2^_1_ = 38.89, *P* < 0.001) and mounts (GLMM: Wald χ^2^_1_ = 69.45, *P* < 0.001) on Day 1 than Day 2. Lastly, we examined opposite-sex strength scores derived from social networks quantifying shared shelter use patterns and found that previously isolated versus socialized males did not differ in their strength of female associations (*P*_*rand*_ = 0.62; Fig. 2d,e, Supplementary Figs. S1 and S4a). Detailed results for all the statistical models we ran can be found in the Supplementary Materials.

### Experiment 2: Effect of social experience on male sexual competency, controlling for insemination status

**Fig. 3.**
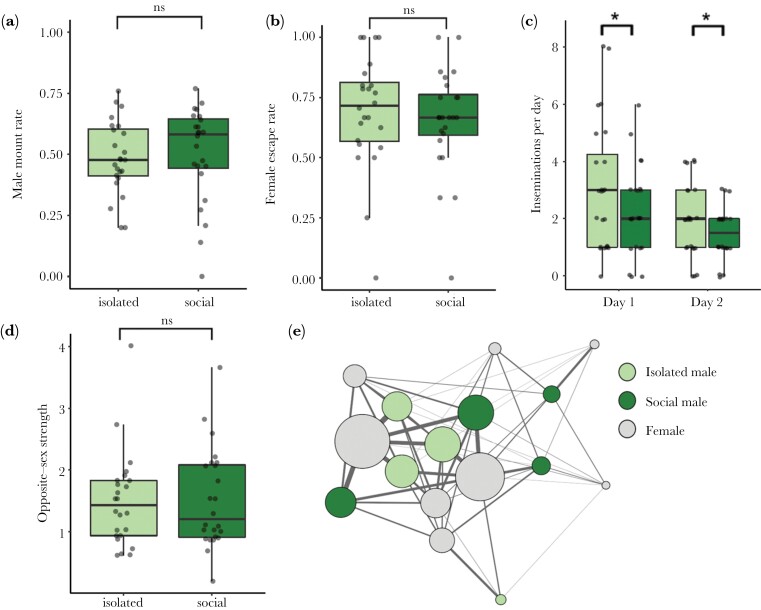
(a) Proportion of mounts males directed towards other males, (b) rate of females successfully evading insemination attempts, (c) insemination rate, and (d) opposite-sex association strength for isolated (*N* = 24) vs. social (*N* = 24) males. (e) Weighted opposite-sex social association network from one replicate based on patterns of shared shelter use. Node color corresponds to individual treatment. Edge width represents the strength of association between opposite-sex dyads, and node size corresponds to opposite-sex strength (total sum of edge weights). Social networks for all 6 replicates are depicted in Supplementary Fig. S2.

In Experiment 1, social experience encompassed insemination status since isolated males were completely restricted from access to females and thus entered the test phase as virgins. Experiment 2 differed from Experiment 1 in that we roughly equalized insemination status of males from the two treatments using controlled mating trials. Again, we did not detect differences in social vs. isolated males’ propensity to mount other males (GLMM: Wald χ^2^_1_ = 0.31, *P* = 0.58; Fig. 3a) and ability to prevent females from escaping mounts (GLMM: Wald χ^2^_1_ = 0.32, *P* = 0.57; Fig. 3b). However, despite us controlling for insemination status, isolated males secured significantly more inseminations compared to social males across the entire observation phase (Mean ± SE: isolated = 2.52 ± 0.29 inseminations per day, social = 0.87 ± 0.18 inseminations per day; GLMM: Wald χ^2^_1_ = 4.52, *P* < 0.05; Fig. 3c). Therefore, we once again tested whether isolated males performed more mounts overall to examine differences in sexual motivation. Again, we found a significant treatment by day interaction for mounts performed (GLMM: Wald χ^2^_1_ = 7.95, *P* < 0.01) driven by isolated males performing more mounts than socialized males on Day 1 (*t* = 3.32, *P* < 0.01; Mean ± SE: isolated = 30.67 ± 3.26, social = 22.00 ± 2.39) but not Day 2 (*t* = 1.42, *P* = 0.16; Mean ± SE: isolated = 24.12 ± 4.38, social = 19.38 ± 1.58). Like in Experiment 1, we again detected significant day effects, with more inseminations (GLMM: Wald χ^2^_1_ = 9.44, *P* < 0.01) and mounts (GLMM: Wald χ^2^_1_ = 44.87, *P* < 0.001) occurring on Day 1. Lastly, our social network analyses revealed that previously isolated versus socialized males did not differ in how strong their social associations were with females (*P*_*rand*_ = 0.89; Fig. 3d,e, Supplementary Figs. S2 and S4b).

### Experiment 3: Effect of social experience on female sexual competency, controlling for insemination history

**Fig. 4.**
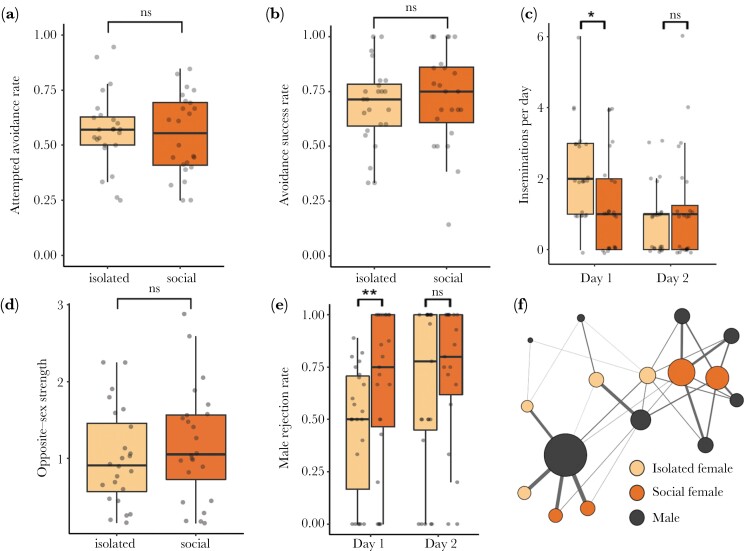
(a) Rate of attempting to avoid mounts, (b) avoidance success rate, (c) insemination rate, (d) opposite-sex association strength, and male rejection rate measured for isolated (*N* = 24) vs. social (*N* = 24) females. (f) Weighted opposite-sex social association network from a one replicate based on patterns of shared shelter use. Node color corresponds to individual treatment. Edge width represents the strength of association between opposite-sex dyads, and node size corresponds to opposite-sex strength (total sum of edge weights). Social networks for all 6 replicates are depicted in Supplementary Fig. S3.

We did not detect an effect of social experience on females’ propensity to attempt evading mounts (GLMM: Wald χ^2^_1_ = 0.11, *P* = 0.75; Fig. 4a), females’ ability to successfully evade males (GLMM: Wald χ^2^_1_ = 0.33, *P* = 0.56; Fig. 4b), or females’ opposite-sex association strength (*P*_*rand*_ = 0.40; Fig. 4d,f, Supplementary Figs. S3 and S4c). However, we did find that females from the isolated treatment were inseminated more than females from the social treatment on Day 1 (*t* = 2.37, *P* < 0.05; Mean ± SE: isolated = 2.21 ± 0.27, social = 1.29 ± 0.29; Fig. 4c) but not Day 2 (*t* = −0.882, *P* = 0.38; Mean ± SE: isolated = 0.83 ± 0.20, social = 1.08 ± 0.32; Fig. 4c). To explore the mechanism driving this increased insemination rate for isolated females on Day 1, we examined number of mounts received by females and found no evidence of males mounting previously isolated females at higher rates than previously socialized females (GLMM: Wald χ^2^_1_ = 0.36, *P* = 0.55; Mean ± SE: isolated = 8.40 ± 1.65, social = 7.81 ± 1.37). Instead, we found that on Day 1, socially housed females faced higher rates of rejection by males measured by proportion of mounts that males terminated for each female (*t* = −0.84, *P* < 0.01; Fig. 4e).

## Discussion

In Experiment 1, we examined how realistic differences in social experience, which encompass differences in mating history, influence various measures of male sexual competency. We found that males provided with social experience did not exhibit improved sexual performance in terms of how often they mounted females as opposed to other males, their abilities to overcome female evasion attempts, and the strength of their opposite-sex social associations (Fig. 2). However, on the first day of the test phase, previously isolated males successfully inseminated females at higher rates compared to previously socialized males. We hypothesized that this difference in the insemination rate was driven by elevated rates of sexual motivation, which was supported by our finding that males from the isolated treatment displayed higher rates of mounting on Day 1 as well. Therefore, we next conducted Experiment 2 to assess the effects of social experience on male sexual behavior while controlling for the effect of sexual deprivation. Once again, we found no experience effects on our various sexual performance metrics (Fig. 3). Yet, to our surprise, isolated males still mounted and inseminated females at higher rates compared to socially housed males. Thus, our findings from Experiments 1 and 2 suggest that, regardless of their insemination history, male bed bugs that experience social isolation during early adulthood display elevated levels of sexual pursuit and motivation leading to increased insemination success compared to group-housed males. These effects of isolation were short-lived as we only documented differences in inseminations and mounts performed on Day 1, most likely because significantly more sexual interactions occurred on Day 1. Alternatively, the effects of social isolation may have been relatively transient because once introduced to a group, previously isolated individuals rapidly gained social experience.

In natural infestations, which can range from a few to thousands of individuals, bed bugs are typically found in mixed-sex aggregations (Johnson 1941; Reinhardt and Siva-Jothy 2007). They are also strongly attracted to the social cues of conspecifics (Levinson and Bar Ilan 1971; Siljander et al. 2007; Yan and Dukas 2022). Therefore, though we provided isolated males with an insemination opportunity each day, the lack of conspecifics and social cues of other bed bugs in isolated males’ housing arenas may have led to perceived mate scarcity. Perceived low mate availability is known to alter courtship, aggression, mate-guarding behavior, and intra-sexual competition in a variety of species (Emlen and Oring 1977; Colwell and Oring 1988; Mitani et al. 1996; Weir et al. 2011; Wacker et al. 2013). In species where the social landscape can be highly variable, it would be beneficial for males to adjust their mating strategies when perceived mate availability is low and to increase sexual pursuit intensity and persistence when encountering potential reproductive opportunities. On the other hand, when mate availability is perceived to be abundant, males may adopt a more conservative mating strategy where they expend their energy, sperm, and seminal fluid reserves more prudently, placing greater investment into somatic maintenance or higher-quality reproductive opportunities. This explanation is supported by the fact that males of many species, including bed bugs, are known to experience sperm and/or seminal fluid depletion (Birkhead 1991; Preston et al. 2001; Wedell et al. 2006; Linklater et al. 2007; Reinhardt et al. 2011) and exhibit choosiness based on female mating status (Cook and Gage 1995; Wedell 1992; Yan et al. under review).

The higher rates of mounting and insemination seen in previously isolated males could also be driven by decreased sexual motivation and sexual pursuit intensity in social males as a response to female rejection. Similar experience effects are well-documented in other species. For example, courtship conditioning is a phenomenon observed in fruit flies (*Drosophila melanogaster*) where exposure to mated, unreceptive females supresses male courtship behavior even once the males are introduced to receptive virgin females (Siegel and Hall 1979; Raun et al. 2021). These experience-based shifts in sexual behavior are mediated by both behavioral and chemical cues (Siwicki et al. 2005; Dukas and Scott 2015). In bed bugs, females are known to display distinct avoidance behaviors, and males can also readily differentiate between recently mated and virgin females even based on only residual social cues from previously occupied shelters (Yan and Dukas 2022). Therefore, the lower mount and insemination rates seen in our socially housed males may suggest that encountering clear rejection signals from females decreases the probability of males investing energy into pursuing potential mates. Further experiments that assess the long-term fitness consequences of such decreased mount and insemination rates are needed to assess whether these responses to rejection are evolutionarily adaptive.

A third, non-mutually exclusive explanation for why isolated males secured a greater number of inseminations is that the presence of rival males during the experience phase caused social males to shift their reproductive investment into post-copulatory traits to account for sperm competition, thus resulting in decreased pre-copulatory success rates. Cues of rivals are known to induce plastic shifts in mating behaviors and reproductive tactics in males of many species (Gross 1996; Bretman et al. 2010, 2011; Auld et al. 2015; Noguera 2019). Furthermore, males that can alter their pursuit behavior according to local levels of competition are predicted to outperform rival males with fixed pursuit strategies (Fawcett and Johnstone 2003; Härdling and Kokko 2005). However, it is worth nothing that currently, very little is known about post-copulatory sexual selection mechanisms in bed bugs. To disentangle the potential effects of perceived low mate availability, experience with female rejection, and presence of rival males, future experiments could expose focal individuals to either only males or only females and assess the relative importance of experience with same versus opposite sex conspecifics.

Unlike in other species (Dukas 2004; Hoefler et al. 2010; Jordan & Brooks 2012), experience did not narrow males’ range of pursuit towards receptive individuals as males from both treatments generally mounted other males as often as they did females. This lack of improvement supports previous assertions that male bed bugs are incapable of discriminating between male and female conspecifics prior to physical contact (Rivnay 1933; Siva-Jothy 2006). Because bed bugs are nocturnal and inhabit tight crevices with patchy distributions of conspecifics, opportunities for pre-copulatory mate assessment may be limited, thus resulting in high rates of same-sex mountings. Red flour beetles (*Tribolium castaneum*) similarly inhabit dark environments and exhibit high rates of male-male sexual behavior (Levan et al. 2009). However, social experience does reduce males’ tendency to display same-sex behavior in flour beetles (Martin et al. 2015). Importantly, male–male pairings in flour beetles often involve the transfer of a spermatophore and, therefore, require considerable energetic investment (Martin et al. 2015). Since bed bugs do not appear to perform any courtship behaviors and rarely inseminate other males, the time and energy costs associated with directing sexual pursuit toward other males are minimal, especially compared to species with extensive courtship. In such cases where males do not display courtship behavior, there may be little to no benefit in possessing strong sex discrimination mechanisms prior to mounting.

We similarly did not find experience effects on males’ capabilities of overcoming female avoidance. It is, therefore, possible that, at least in bed bugs, the ability to prevent females from resisting mounts is a fixed trait determined by anatomical or physiological differences that affect mounting speed or grasping strength as opposed to a behaviorally plastic trait. Interestingly, sexual experience similarly does not appear to impact a male’s ability to successfully mate in eastern mosquitofish (*Gambusia holbrooki*), another species well-known for its coercive mating system (Iglesias-Carrasco et al. 2019). Currently, it remains unclear why some species show strong effects of experience on sexual competence while others do not. One possibility is that experience may be more beneficial to males in mating systems that involve complex suites of behaviors related to courtship or competition compared to systems characterized by high sexual conflict and persistent harassment.

In females, we similarly did not detect differences between isolated and social individuals’ propensity to avoid mounts and success in avoiding mounts. While social females were inseminated more than isolated females on Day 1, our additional analyses revealed that this difference was driven by males terminating mounts directed at social females at higher rates instead of social females displaying increased sexual competency. Most likely, chemical cues indicating high previous sexual encounter rates made the social females less attractive to males. We had expected social females to attempt evading mounts at higher rates due to exposure to frequent harassment from males during their experience phase and the fact that sexual harassment is known to negatively impact the fitness of females in bed bugs and many other species (Helinski and Harrington 2012; Okada et al. 2017; Réale et al. 1996; Sakurai and Kasuya 2008; Saveer et al. 2021; Yan et al. under review). Furthermore, while the ability to successfully evade males could be constrained by physical or physiological limitations and, therefore, be behaviorally inflexible, we have previously demonstrated that female bed bugs increase male evasion after they experience a few inseminations (Yan et al. under review). Thus, the lack of difference in attempted avoidance rate between previously isolated versus social females remains puzzling. Moreover, our video recordings of social females during the experience phase revealed fairly frequent male encounters (12.5 times per dark period) and mount rates (6.25 times per dark period), meaning that social females received considerably more experience with males compared to females in the isolated treatment. Nonetheless, it remains possible that even higher rates of encountering males and receiving harassment are necessary to generate clear differences in females’ propensity or ability to evade males. Future experiments can resolve these questions by manipulating the population density, sex ratio, arena size, or availability of refuges to examine the effects of experience under variable environmental conditions.

Lastly, we found that across all 3 experiments, experience did not predict an individual’s position in opposite-sex association networks. Past research has often reported major social deficits in individual animals that have previously experienced social isolation (Harlow et al. 1965; Lihoreau et al. 2009; Hesse and Thünken 2014). However, a majority of these studies manipulated the rearing conditions of focal animals throughout their developmental period while the bed bugs in our experiments were all reared socially in the pre-adult stages and then assigned to isolated or social conditions during early adulthood. The consequences of social deprivation may vary greatly depending on the timing and duration of isolation throughout an animal’s lifetime. As such, it remains entirely possible that juvenile social experience plays a role in adult social and sexual behavior in bed bugs. Future studies should seek to determine the most critical periods for social enrichment to further our understanding of experience-based effects.

Because the edges of our social networks represented patterns of shared shelter use between males and females, we hypothesized that strong connections to opposite-sex conspecifics would reflect males’ ability to locate and retain continued access to insemination opportunities and females’ inability to find refuge from males. Social network centrality has been found to be associated with various fitness measures across several taxa (Sabol et al. 2020; Beck et al. 2021; Turner et al. 2021). Moreover, the degree and strength of opposite-sex associations have been strongly tied to reproductive fitness in males (Dunning et al. 2023) and a number of copulations in females (McDonald et al. 2019). We had predicted that bed bugs would exhibit a similar link between social network position and various measures of reproductive success since access to mates is one of the major proposed benefits of group formation in animals. However, to our surprise, higher opposite-sex network strength did not predict a number of inseminations in bed bugs (Yan and Dukas, unpublished data). As a result, despite bed bugs exhibiting sociality via aggregation formation and chemical communication using various pheromones, our current understanding of how or whether network position in social association networks affects reproductive success remains poorly understood. Overall, to advance our understanding of animal sociality, we have to further examine the relationship between social connectedness and fitness in moderately social species.

## Supplementary Material

arae030_suppl_Supplementary_Materials

## Data Availability

Analyses reported in this article can be reproduced using the data provided by Yan et al (2024).
