# Supplementary material for: Sexual conflict and social networks in bed bugs: effects of social experience
Source: Behav Ecol. 2024 Apr 17;35(3):arae030. doi: 10.1093/beheco/arae030 (PMC11059254; doi:10.1093/beheco/arae030)
Supplement: arae030_suppl_Supplementary_Materials [file arae030_suppl_supplementary_materials.docx]

**
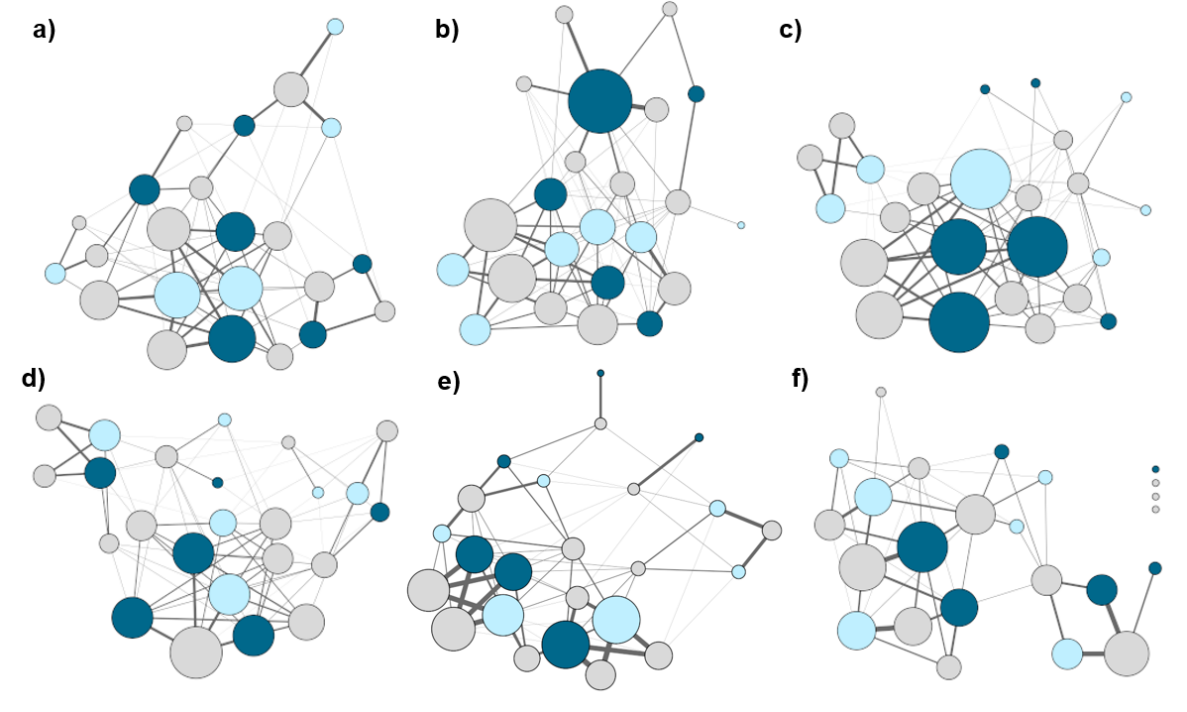
SUPPLEMENTARY INFORMATION**

**Figure S1. Experiment 1 networks.** Weighted opposite-sex social association network from all six replicates of Experiment 1 based on patterns of shared shelter use. Node colour corresponds to individual treatment (light blue = isolated male, dark blue = social male, grey = female). Edge width represents the strength of association between opposite-sex dyads and node size corresponds to opposite-sex strength (total sum of edge weights). Nodes with no connections represent individuals that were never observed sharing a shelter with a member of the opposite sex.

| **Experiment 1:** Effect of social experience on male sexual competency | | | | | | |
| --- | --- | --- | --- | --- | --- | --- |
| **Response variable** | **Parameter** | **Estimate** | **SE** | **Wald χ^2^** | ***d.f.*** | **p-value** |
| Rate at mounting other males vs. other females | Treatment | 0.1453 | 0.2122 | 0.4688 | 1 | 0.4936 |
|  | Day | 0.5411 | 0.1730 | 9.7841 | 1 | **0.0018** |
|  | Treatment:Day | -0.5421 | 0.2609 | 4.3163 | 1 | **0.0378** |
| Rate at which female successfully escaped mounts | Treatment | 0.0765 | 0.3337 | 0.2586 | 1 | 0.6111 |
|  | Day | 0.5356 | 0.3770 | 0.5626 | 1 | 0.4532 |
|  | Treatment:Day | -0.6904 | 0.5446 | 1.6069 | 1 | 0.2049 |
| Number of inseminations performed | Treatment | -0.5298 | 0.1623 | 10.662 | 1 | **0.0011** |
|  | Day | -1.3055 | 0.2094 | 38.888 | 1 | **< 0.001** |
|  | Treatment:Day | 0.5953 | 0.3086 | 3.7200 | 1 | **0.0538** |
| Number of mounts performed | Treatment | -0.5802 | 0.1388 | 17.481 | 1 | **< 0.001** |
|  | Day | -1.2008 | 0.1441 | 69.452 | 1 | **< 0.001** |
|  | Treatment:Day | 0.7101 | 0.2086 | 11.586 | 1 | **< 0.001** |

**Table S1. Mixed-effect model results for Experiment 1.**


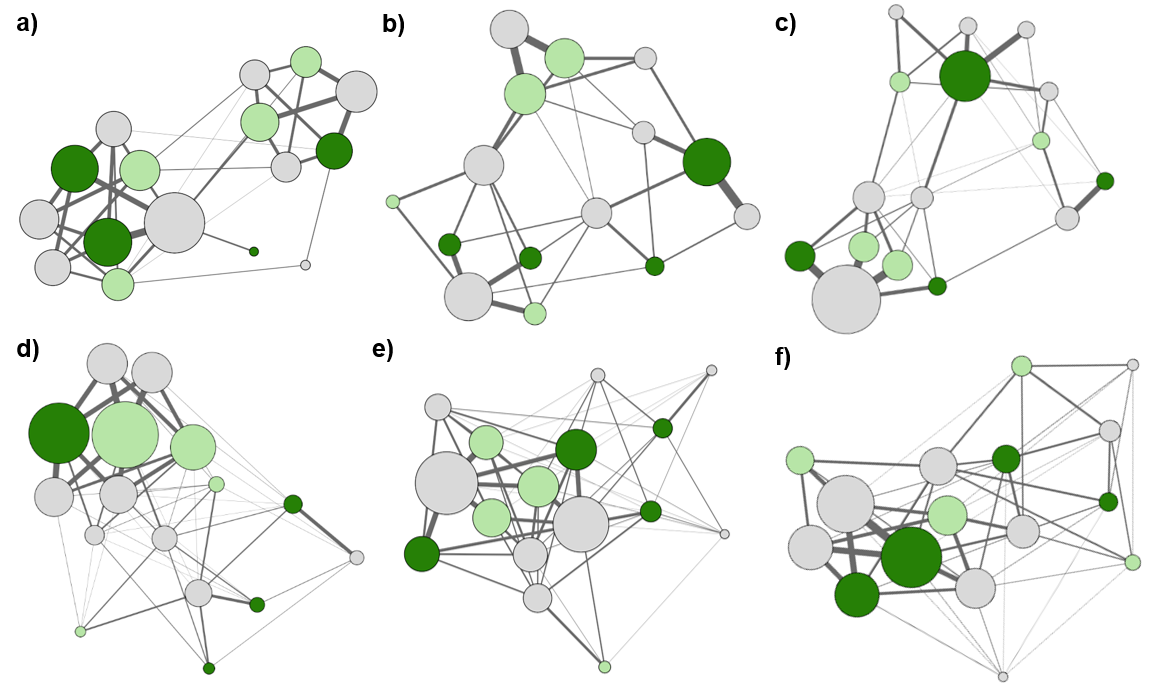
**Figure S2. Experiment 2 networks.** Weighted opposite-sex social association network from all six replicates of Experiment 2 based on patterns of shared shelter use. Node colour corresponds to individual treatment (light green = isolated male, dark green = social male, grey = female). Edge width represents the strength of association between opposite-sex dyads and node size corresponds to opposite-sex strength (total sum of edge weights).

**Table S2. Mixed-effect model results for Experiment 2.**

| **Experiment 2:** Effect of social experience on male sexual competency, controlling for insemination status | | | | | | |
| --- | --- | --- | --- | --- | --- | --- |
| **Response variable** | **Parameter** | **Estimate** | **SE** | **Wald χ^2^** | ***d.f.*** | **p-value** |
| Rate at mounting other males vs. other females | Treatment | 0.0623 | 0.2190 | 0.3112 | 1 | 0.5770 |
|  | Day | 0.0312 | 0.1182 | 0.7897 | 1 | 0.3742 |
|  | Treatment:Day | 0.1165 | 0.1824 | 0.4079 | 1 | 0.5230 |
| Rate at which female successfully escaped mounts | Treatment | -0.0766 | 0.3540 | 0.3213 | 1 | 0.5708 |
|  | Day | 0.1294 | 0.2909 | 1.5911 | 1 | 0.2072 |
|  | Treatment:Day | 0.3875 | 0.4622 | 0.7031 | 1 | 0.4017 |
| Number of inseminations performed | Treatment | -0.3151 | 0.1790 | 4.5195 | 1 | **0.0335** |
|  | Day | -0.4539 | 0.1865 | 9.4445 | 1 | **0.0021** |
|  | Treatment:Day | 0.04845 | 0.2848 | 0.0290 | 1 | 0.8649 |
| Number of mounts performed | Treatment | -0.4182 | 0.1259 | 11.015 | 1 | **< 0.001** |
|  | Day | -0.3625 | 0.0539 | 44.871 | 1 | **< 0.001** |
|  | Treatment:Day | 0.2355 | 0.0832 | 7.9510 | 1 | **0.0048** |

| **Experiment 3:** Effect of social experience on female sexual competency, controlling for insemination status | | | | | | |
| --- | --- | --- | --- | --- | --- | --- |
| **Response variable** | **Parameter** | **Estimate** | **SE** | **Wald χ^2^** | ***d.f.*** | **p-value** |
| Proportion of mounts females attempted to avoid | Treatment | -0.3010 | 0.2268 | 0.1054 | 1 | 0.7454 |
|  | Day | -0.1356 | 0.2142 | 0.4925 | 1 | 0.4828 |
|  | Treatment:Day | 0.5434 | 0.3107 | 3.0598 | 1 | 0.0802 |
| Proportion of avoid attempts that were successful | Treatment | 0.42152 | 0.40521 | 0.3331 | 1 | 0.5639 |
|  | Day | -0.01957 | 0.33191 | 0.8649 | 1 | 0.3524 |
|  | Treatment:Day | -0.47651 | 0.50013 | 0.9078 | 1 | 0.3407 |
| Number of inseminations received | Treatment | -0.5363 | 0.2261 | 5.6258 | 1 | **0.0176** |
|  | Day | -0.9746 | 0.2624 | 13.791 | 1 | **< 0.001** |
|  | Treatment:Day | 0.8364 | 0.3717 | 4.5697 | 1 | **0.0325** |
| Number of mounts received | Treatment | -0.1044 | 0.2124 | 0.3609 | 1 | 0.5480 |
|  | Day | -0.2520 | 0.2021 | 2.7454 | 1 | 0.0975 |
|  | Treatment:Day | 0.0116 | 0.3004 | 0.0015 | 1 | 0.9691 |
| Rate of rejection by males | Treatment | 0.8444 | 0.3020 | 2.7066 | 1 | 0.0999 |
|  | Day | 1.1844 | 0.3246 | 7.3355 | 1 | **0.0068** |
|  | Treatment:Day | -1.1296 | 0.4619 | 5.9818 | 1 | **0.0144** |

**
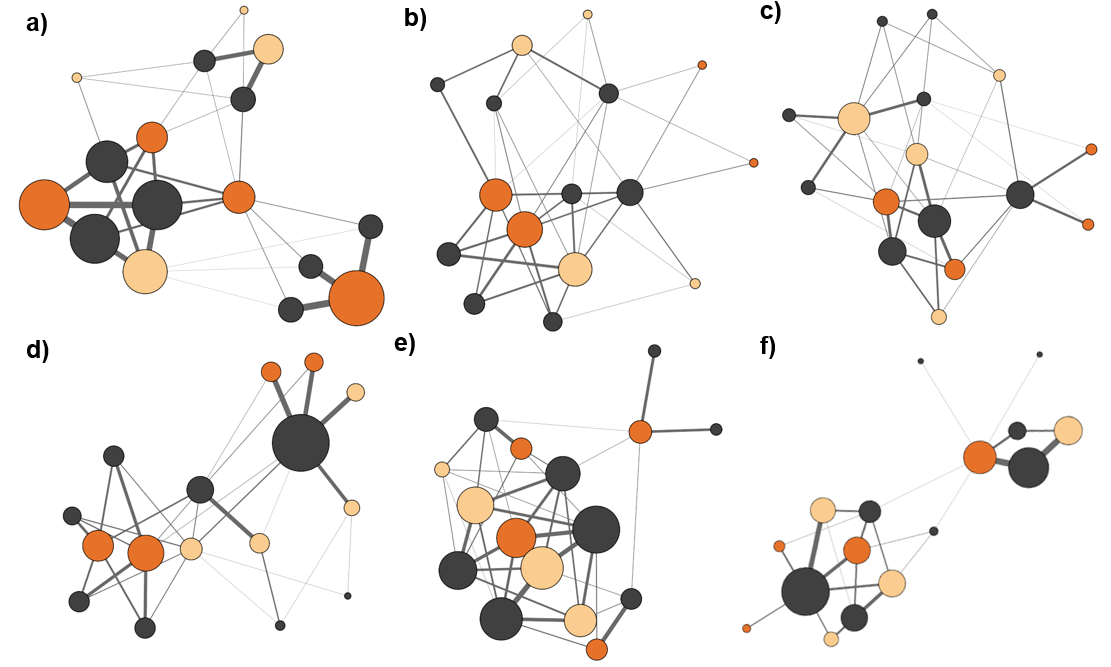
Figure S3. Experiment 3 networks.** Weighted opposite-sex social association network from all six replicates of Experiment 3 based on patterns of shared shelter use. Node colour corresponds to individual treatment (light orange = isolated female, dark orange = social female, black = male). Edge width represents the strength of association between opposite-sex dyads and node size corresponds to opposite-sex strength (total sum of edge weights).

**Table S3. Mixed-effect model results for Experiment 3.**


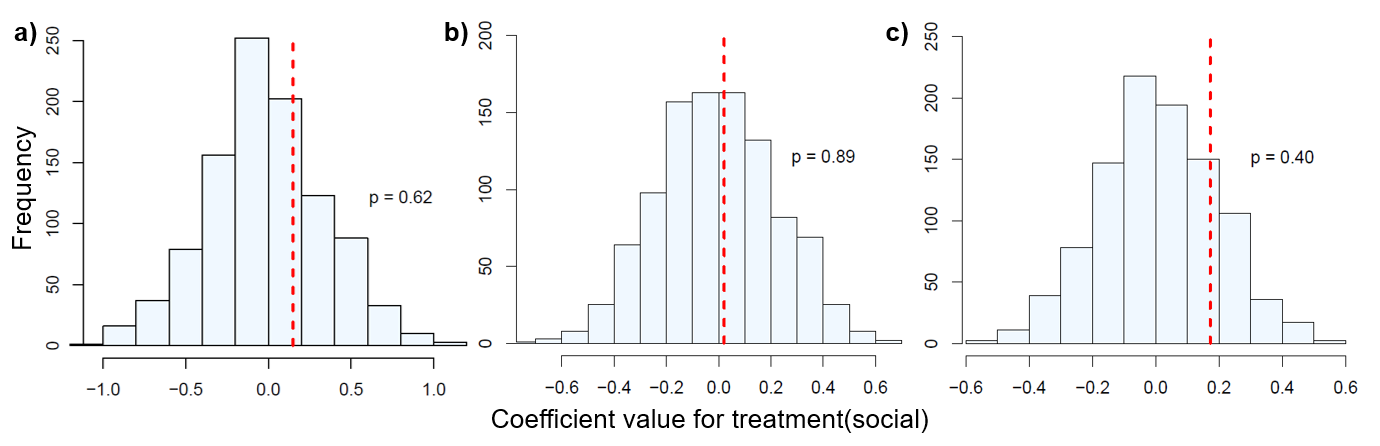
**Figure S4.** Null distributions of LMM model coefficients representing the effect of treatment on opposite-sex strength for (a) Experiment 1, (b) Experiment 2, and (c) Experiment 3. Null distributions for each treatment are the result of 1000 node-label network randomizations. Red dashed lines are model coefficients representing the observed effect of treatment on opposite-sex strength for each experiment.
